# Gastric lymphoma complicated by phlegmonous gastritis and Guillain–Barré syndrome: A case report

**DOI:** 10.1097/MD.0000000000020030

**Published:** 2020-05-01

**Authors:** Kodai Kuriyama, Yuki Koyama, Kazuma Tsuto, Natsuko Tokuhira, Hiroaki Nagata, Ayako Muramatsu, Muneo Oshiro, Yoshiko Hirakawa, Toshiki Iwai, Hitoji Uchiyama

**Affiliations:** a Department of Hematology; b Department of Gastroenterology; c Department of Neurology and Stroke Treatment; d Department of Anesthesiology, Japanese Red Cross Kyoto Daiichi Hospital, Kyoto, Japan.

**Keywords:** diffuse large B-cell lymphoma, gastric lymphoma, Guillain–Barré syndrome, phlegmonous gastritis

## Abstract

**Introduction:**

Complications such as severe infection may occur during the chemotherapy of malignant lymphoma. Phlegmonous gastritis (PG) is a rare acute bacterial infection associated with high mortality, requiring early diagnosis, and prompt management. In addition, Guillain–Barré syndrome (GBS) occasionally requires early treatment and intensive care management due to the occurrence of severe neuropathy and respiratory failure.

**Patient concerns:**

A 70-year-old male was diagnosed with primary gastric diffuse large B-cell lymphoma (DLBCL) after the detection of several polypoid tumors with ulcers. The patient underwent chemotherapy for DLBCL and exhibited adverse effects (i.e., fever, vomiting, epigastric pain, and neutropenia). Computed tomography indicated widespread thickening in the gastric wall. Furthermore, approximately 2 weeks later, the patient presented with gradual symmetric lower extremity weakness and respiratory failure due to paralysis of the respiratory muscle.

**Diagnoses:**

DLBCL was diagnosed through a gastric tumor biopsy. On the basis of the computed tomography findings, a culture of gastric juice, nerve conduction studies, and clinical symptoms, this case of gastric lymphoma was complicated with PG and GBS.

**Interventions:**

The patient was treated with antimicrobial therapy and administration of granulocyte colony-stimulating factor for PG, and with intravenous immunoglobulin and intensive care management for GBS.

**Outcomes:**

Despite the aggressive progress of the condition, the patient improved without relapse of DLBCL.

**Conclusion:**

PG was regarded as a precedent infection of GBS. In this article, we present the first reported case of gastric lymphoma complicated with PG and GBS.

## 1 Introduction

Non-Hodgkin lymphoma (NHL) is a hematopoietic malignancy disorder involving the lymph nodes and extranodal sites such as the gastrointestinal tract.^[1]^ The most common subtype of NHL is diffuse large B-cell lymphoma (DLBCL). The standard treatment for DLBCL is chemotherapy and radiotherapy.^[2,3]^ However, chemotherapy for NHL is associated with the occurrence of complications (e.g., severe infection).

Phlegmonous gastritis (PG) is an uncommon disease characterized by a severe bacterial infection of the gastric wall.^[4]^ PG is linked to a high mortality rate because early diagnosis remains difficult.^[5–9]^ In most previous reports, gastric mucosal injury due to gastric cancer or disorder is regarded as the main etiology of PG.^[10–12]^ In cases suspected of PG, early diagnosis using various modalities and prompt antimicrobial therapy are required. For those in whom conservative treatment is ineffective, surgical treatment should be performed immediately to prevent worsening of the patient's condition.

In this article, we describe a rare case of PG complicated with gastric lymphoma while undergoing treatment with chemotherapy. Interestingly, this severe gastric infection caused severe Guillain–Barré syndrome (GBS). To the best of our knowledge, this is the first case report of gastric lymphoma complicated with PG and GBS.

## 2 Case report

**Figure 1:**
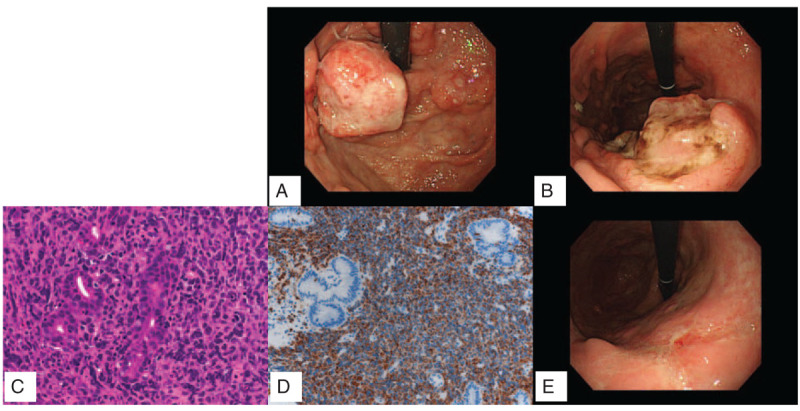
(A) (B) Upper gastrointestinal endoscopy indicating the presence of several polypoid tumors with ulcers at the lesser curvature of the gastric body and gastric cardiac region. (C) Hematoxylin–eosin stain of the gastric tumor at × 40 magnification. (D) CD20 immunohistochemical staining of the gastric tumor at × 20 magnification. (E) Upper gastrointestinal endoscopy showing the absence of polypoid tumors after 3 cycles of chemotherapy. (B and E are identical angles.).

The patient was a 70-year-old male admitted to our hospital due to a gastric tumor detected through medical examination. Upper gastrointestinal endoscopy indicated the presence of several polypoid tumors with ulcers at the lesser curvature of the gastric body and the gastric cardiac region (Fig. 1A and 1B). Histopathological assessment of the biopsied gastric tumor identified diffuse infiltration of abnormal large lymphoid cells (Fig. 1C). These cells were positive for CD20 (Fig. 1D) and BCL-6, and negative for CD3, CD5, CD10, CD43, MUM1, and BCL-2. Further examination using ^18^F-labeled fluorodeoxyglucose positron emission tomography–computed tomography (CT) indicated that the uptake of ^18^F-labeled fluorodeoxyglucose was high only in the stomach.

On the basis of these results, the patient was diagnosed with primary gastric DLBCL (stage IA, International Prognostic Index; low risk).^[13–15]^ We planned 6 cycles of systemic R-CHOP chemotherapy (i.e., rituximab, cyclophosphamide, doxorubicin, vincristine, and prednisolone) – the standard chemotherapy for DLBCL.^[16,17]^ He received the first cycle of treatment in the hospital without developing serious adverse events. The remaining cycles were received in the outpatient department. After the third cycle, upper gastrointestinal endoscopy showed that the tumor lesions had disappeared and slight ulcers remained. Hence, we concluded that the patient achieved almost complete remission (Fig. 1E).

**Figure 2:**
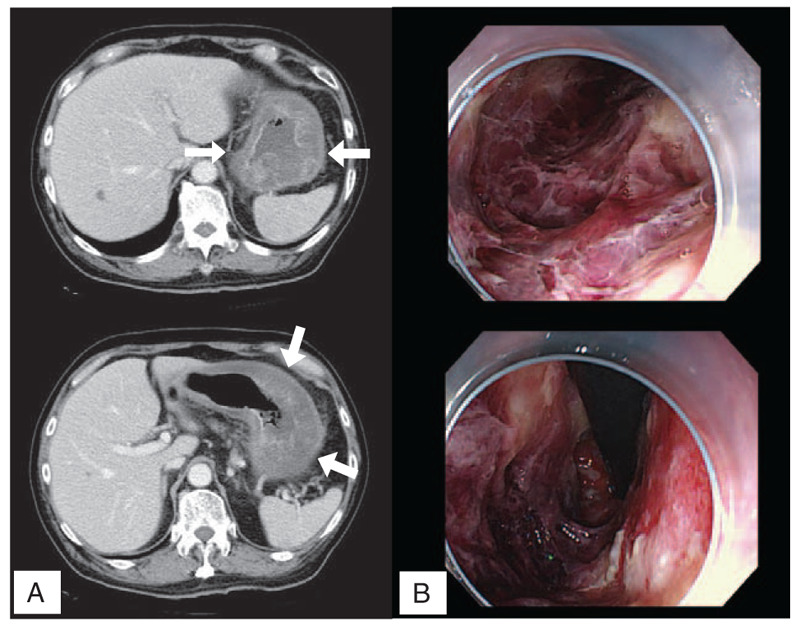
(A) Contrast-enhanced computed tomography image at the onset showing a diffuse thickened gastric wall (arrows). (B) Upper gastrointestinal endoscopy showing extensive gastric deep ulcerations with necrotic tissue and an edematous mucosa.

However, on day 8 of the fifth cycle of chemotherapy, the patient was transferred to the emergency department of our hospital by ambulance because of fever, vomiting, and epigastric pain. Examination indicated that the temperature was 39.0°C, the blood pressure was 116/60 mm Hg, the heart rate was 92 beats per minute, and the oxygen saturation was 96% while the patient was breathing ambient air. The epigastrium was tender without abdominal guarding, rebound tenderness, or stool loading. The remainder of the examination was unremarkable. Laboratory test results indicated a decrease in the white blood cell count to 0.39 × 10^9^/L (reference range: 4.0–8.0 × 10^9^/L), neutropenia (neutrophils 7.7% [reference range: 40%–72%]), and a very high inflammatory status (C-reactive protein: 30.5 mg/dL [reference range: 0–0.3 mg/dL]). Leukocytopenia was regarded as myelosuppression due to chemotherapy. The levels of electrolytes in the blood as well as the renal and liver function tests were unremarkable. CT of the abdomen – performed after the administration of intravenous contrast material – demonstrated widespread thickening in the gastric wall without signs of free air (Fig. 2A).

The presence of a severe infection was considered, and meropenem was intravenously administered along with fasting management and urgent hospitalization. On day 2 of hospitalization, upper gastrointestinal endoscopy showed extensive gastric ulceration with necrotic tissue and edematous mucosa (Fig. 2B). At that time, we could not perform biopsy because we could not clearly observe the intragastric state. On day 9 of hospitalization, re-examination using upper endoscopy and histopathological assessment of the biopsied gastric lesion did not reveal findings of lymphoma relapse. Although cultures of blood and the gastric biopsies were negative, *Pseudomonas aeruginosa* was isolated from a culture of gastric juice. On the basis of these results and clinical features, we diagnosed the patient with severe PG.

Following the administration of meropenem, proton pump inhibitors, and granulocyte colony-stimulating factor, his general condition gradually and steadily improved. However, on day 13 of hospitalization, he presented with gradual symmetric lower extremity weakness and was unable to ambulate. Moreover, he reported dysphagia and mis-swallowing. On day 24 of hospitalization, he presented with respiratory failure due to paralysis of the respiratory muscle. Arterial blood gas analysis indicated significant hypercarbia and acidosis. He was transferred to the intensive care unit for respirator management because his consciousness rapidly worsened due to CO_2_ narcosis. Neurological examination indicated that the deep tendon reflexes were absent. Lumbar puncture indicated normal cell count and protein levels. Examination of the cerebrospinal fluid through polymerase chain reaction for herpes simplex virus type 1/2, varicella zoster virus, and human herpesvirus-6 DNA was negative. Nerve conduction studies showed that the amplitude of compound muscle action potentials was reduced and the incidence of the F wave was significantly reduced in the motor nerves (ulnar and median). In the sensory nerves (ulnar, median, and radial), the amplitude of sensory nerve potentials was in the lower limits of normal. Furthermore, serum antiganglioside antibodies were negative. Collectively, these clinical signs and symptoms suggested the diagnosis of acute motor axonal neuropathy – a subtype of GBS.

Subsequently, the patient was treated with intravenous immunoglobulin (0.4 kg/mg/d) for 5 days. In addition, he was managed with intensive care and elaborate rehabilitation. He gradually recovered from extremity weakness and respiratory muscle paralysis and was released from respirator management on day 27 of hospitalization. Later, he underwent long-term rehabilitation at another hospital. Six months later, he was able to walk without assistance and was followed up at the outpatient department without findings of DLBCL relapse.

## 3 Discussion

Complications such as severe infection may occur during the chemotherapy of malignant lymphoma. PG is a rare yet often fatal acute pyogenic infection. Therefore, early recognition and prompt treatment are vitally important. In this article, we report a rare case of severe PG complicated with gastric lymphoma while receiving treatment with chemotherapy. Although this case of severe gastric infection resulted in the development of severe GBS, the patient recovered completely with early treatment intervention and intensive care management.

DLBCL is a hematopoietic malignancy disorder involving the lymph nodes and extranodal sites. Notably, extranodal involvement is considered a poor prognostic factor.^[15,18,19]^ In our case, R-CHOP ± radiotherapy was considered as the treatment strategy because the invasion of lymphoma was only gastric.^[16,17]^ Therefore, it is conceivable that the prognosis of this patient was relatively good. Of note, severe infection with myelosuppression is occasionally observed in patients undergoing R-CHOP chemotherapy.

PG is a rare acute bacterial infection requiring prompt diagnosis and management. Despite the administration of appropriate treatment, the mortality rate associated with PG is very high (approximately 30%–40%).^[5–9]^ Therefore, appropriate examination and antimicrobial therapy should be started early. However, when conservative treatment is ineffective, surgical treatment should be immediately performed to prevent worsening of the patient's condition. PG is characterized by fever and nonspecific gastrointestinal symptoms (i.e., epigastric pain, nausea, vomiting, diarrhea, and hematemesis). Findings highly indicative of PG include a thickened gastric wall through CT and extensive ulceration via upper gastrointestinal endoscopy. This case is consistent with the diagnosis of PG from clinical features and these findings.^[5,9,12]^ The most common causative organisms of PG are *Streptococcus* spp. (approximately 70%), and polymicrobial infection is also common.^[9,20]^ In the present case, blood cultures and cultures of the gastric biopsies were negative. Gastric biopsy findings might be due to delayed biopsy. Gastric biopsy could not be performed during the first upper gastrointestinal endoscopy because we could not clearly observe the intragastric state due to extensive ulceration and necrotic tissue in the gastric region, but it was performed during the second gastrointestinal endoscopy, during which the antibiotic therapy had already started. Therefore, the causative bacteria could not be detected in the culture of gastric biopsy specimen. However, *P. aeruginosa* was isolated from the culture of gastric juice collected during the first gastrointestinal endoscopy. Notably, *P. aeruginosa* has been reported as a causative organism of neutropenic infection.^[21,22]^ In this regard, we considered that the present infection with *P. aeruginosa* led to the development of PG.

Previous studies have identified the risk factors of PG: advanced age, infection, malnutrition, diabetes, immunosuppression, ulcer, achlorhydria, gastric mucosal injury from chronic gastritis, and gastric cancer.^[6,8,23]^ Patients with hematological malignancies are commonly immunocompromized due to prolonged neutropenia, intensive chemotherapy, and the disease itself. Indeed, there are a few reports of PG occurring within days of immunosuppresant therapy.^[10,24]^ However, the clinical condition of patients as well as duration between immunosuppressant therapy and PG differ between these reports. Saito et al reported on a patient with acute lymphoblastic leukemia who developed PG during the neutropenic period due to chemotherapy.^[24]^ Our patient exhibited myelosuppression and gastric mucosal injury during the neutropenic period on day 8 of the fifth cycle of chemotherapy for gastric lymphoma, raising the possibility of a severe infection. *P. aeruginosa* rather than *Campylobacter jejuni* was found in the culture of gastric juice. Therefore, we considered that severe PG was the precedent infection of GBS.

GBS is an acute, inflammatory, immune-mediated polyneuropathy characterized by symmetric extremity muscle weakness and hyporeflexia or areflexia.^[25,26]^ Although the primary causative organism is often unclear, preceding infections such as respiratory or gastrointestinal infections trigger the onset of GBS in two-thirds of the cases. In particular, gastrointestinal infection with *C. jejuni* is associated with the development of acute motor axonal neuropathy.^[27,28]^ Previous reports have described patients with lymphoma who developed GBS. In almost all the cases, the condition was reported as paraneoplastic neurological syndrome.^[29–33]^ Even though the present case did not have serum antiganglioside antibodies or cerebrospinal fluid albuminocytologic dissociation, the patient was diagnosed with GBS on the basis of clinical symptoms and the results of nerve conduction studies. This diagnosis is consistent with GBS diagnostic criteria.^[25,26]^ In the present case, we considered GBS not a paraneoplastic syndrome of lymphoma but a complication of PG. Additionally, the patient developed PG because of myelosuppression and gastric mucosal injury due to the treatment for gastric lymphoma. Therefore, we considered that lymphoma, PG, and GBS were linked and related. To the best of our knowledge, this is the first report of such a case. The patient received intravenous immunoglobulin, intensive care management, and long-term rehabilitation, gradually improving in the long term without sequelae.

In conclusion, we present the first case of gastric lymphoma complicated with PG and GBS. PG is linked to high mortality and requires early diagnosis using various modalities and proper management (e.g., antimicrobial therapy). In addition, GBS occasionally requires early treatment and intensive care management due to the occurrence of severe neuropathy and respiratory failure. Detailed knowledge of the relevant clinical features and imaging findings is indispensable for early diagnosis and treatment, especially in immunocompromized patients such as those undergoing chemotherapy for lymphoma.

## Acknowledgments

The authors would like to thank Enago (www.enago.com) for the English language review.

## Author contributions

**Conceptualization:** Kodai Kuriyama, Yuki Koyama, Kazuma Tsuto, Natsuko Tokuhira, Hiroaki Nagata, Ayako Muramatsu, Muneo Oshiro, Yoshiko Hirakawa, Toshiki Iwai, Hitoji Uchiyama.

**Investigation:** Yuki Koyama, Kazuma Tsuto, Natsuko Tokuhira, Hiroaki Nagata, Ayako Muramatsu, Muneo Oshiro, Yoshiko Hirakawa, Toshiki Iwai, Hitoji Uchiyama.

**Writing – original draft:** Kodai Kuriyama.

**Writing – review & editing:** Kodai Kuriyama.
